# Expression of sirtuins 1 in placenta, umbilical cord, and maternal serum of patients diagnosed with placenta accreta spectrum

**DOI:** 10.1590/1806-9282.20240314

**Published:** 2024-08-16

**Authors:** Irmak Icen Taskin, Sevim Gurbuz, Mehmet Sait Icen, Dilek Cam Derin, Fatih Mehmet Findik, Engin Deveci

**Affiliations:** 1 Inonu University, Faculty of Science and Art, Department of Molecular Biology and Genetics – Malatya, Turkey.; 2 Dicle University, Department of Obstetrics and Gynecology –Diyarbakır, Turkey.; 3 Dicle University, Faculty of Medicine, Department of Histology and Embryology – Diyarbakır, Turkey.

**Keywords:** Umbilical cord, Serum, Placenta accreta, Sirtuin 1

## Abstract

**OBJECTIVE:**

Placenta accreta spectrum (PAS) is defined as the attachment of the placenta to the uterine wall in varying degrees. However, the studies have explored that the underlying molecular mechanisms of the PAS are very limited. Sirtuins 1 (SIRT1) is associated with placental development by controlling trophoblast cell invasion and remodeling of spiral arteries. We aimed to determine the expression level of SIRT1 in placentas, and maternal and umbilical cord serum of patients with PAS.

**METHODS:**

In total, 30 individuals in control, 20 patients in the placenta previa group, and 30 patients in the PAS group were included in this study. The expression levels of SIRT1 in the placentas were determined by Western blot and immunohistochemistry. Serum levels of SIRT1 in maternal and umbilical cord blood were determined by ELISA.

**RESULTS:**

SIRT1 was significantly lower in placentas of the PAS. However, maternal and umbilical cord serum samples were not significantly different between groups.

**CONCLUSION:**

SIRT1 may play an important role in the pathogenesis of the PAS.

## INTRODUCTION

The placenta accreta spectrum (PAS) refers to the excessive trophoblast invasion of part or all of the placenta into the uterine wall's myometrium^1^. It is histopathologically categorized into placenta accreta, placenta increta, and placenta percreta based on the degree of attachment to the myometrium^2^. The frequency of PAS has increased about eight times since the 1970s^3^, and the overall proportion of PAS in recent times had indeed reached 0.91%^4^. PAS is associated with significant maternal morbidity because the post-delivery placenta of the fetus does not spontaneously separate and can lead to severe hemorrhage, frequently leading to an exigency hysterectomy, blood transfusion, and critical care unit admission^5^. Uterine scar, cesarean history, and presence of placenta previa (PP) are the main clinical risk factors^2^. Accreta placentation is the consequence of implantation and placental development in a uterine scar (mainly post C-section in the lower segment) where the myometrium has been replaced by scar tissue and is often extremely thin with loss of the decidua, junctional zone, and spiral artery circulation. Hence, it is recently defined as a disorder of defective decidua and uterine scar dehiscence, and not as a disorder of destructive trophoblast invasion^6^. However, the molecular mechanism underlying PAS is not fully understood.

Sirtuins (SIRT) are a highly conserved NAD^+^-dependent histone deacetylase family and regulate different important cellular pathways such as DNA repair, transcriptional regulation, metabolism, and aging^7,8^. They also have complex roles in either promoting or suppressing epithelial-mesenchymal transition (EMT), and their functional features may largely depend on the cellular context^8^. Sirtuins 1 (SIRT1) is a member of the sirtuins family. It is extensively expressed in the human and mouse placenta and decreased following labor^9^. In the last trimester of pregnancy, the SIRT1 level of the trophoblasts of both the patients with preeclampsia^10^, and in the placenta of the obese mice is decreased^11^. It is also downregulated in advanced maternal age pregnancies^12^. In addition, it has been shown that SIRT1 may be related to placental development by controlling EVT invasion and spiral artery remodeling through modulation of EMT^13^. However, there are no studies on the role of SIRT1 in PAS. Therefore, in this study, we aimed to investigate the expression of SIRT1 in placenta, maternal serum, and umbilical cord serum samples of PAS patients.

## METHODS

### Study design and population

The study includes 30 individuals in control, 20 patients in the PP group, and 30 patients in the PAS group. Patients who applied to the Dicle University Faculty of Medicine, Department of Obstetrics and Gynecology, were included in our study. This study protocol was approved by the Inonu University, Clinical Research Ethics Committee (2020/51), and the study was performed in accordance with the ethical standards as laid down in the 1964 Declaration of Helsinki. Written informed consent was obtained from all the participants. Three groups of patients were formed. The first group consists of patients with no history of cesarean section, uterine intervention, or uterine surgery but who were diagnosed with PP without invasion were included, and this group was called the PP group. In the second group, patients who had at least one previous cesarean section and had PP and invasion were included, and this group was called the PAS group. The third group was the control group, and those with similar demographic features with PP and PAS groups and with no known disease were included. The patients in the control group were formed from patients who delivered by cesarean section. Patients with PP marginalis or inferior placenta, those who underwent surgery before the 24th week of pregnancy, individuals under the age of 18 years, multiple pregnancies, patients with pregnancy complications in the past, thyroid dysfunction, hypertension, epilepsy, those with gestational diabetes mellitus, and those with type 1 and type 2 diabetes mellitus were not included in the study. For preoperative diagnosis, abdominal, transvaginal, and Doppler ultrasonography were used. PAS or PP was defined according to current FIGO consensus guidelines^5^. PAS was diagnosed with the pathology results. Age, number of pregnancies, and gestational weeks of the patients were compatible with the control group.

### Collecting placental and serum samples

Placental tissues containing villous and extravillous trophoblasts, the fibrinoid layer, and the basal plate layer were obtained in all groups. They were collected after cesarean section and flash frozen using liquid nitrogen and stored at −80° until Western blot analysis. For ELISA, maternal peripheral venous blood was drawn before the administration of anesthesia in patients who underwent general anesthesia and before spinal anesthesia in patients who underwent spinal surgery. Umbilical cord blood was taken from the umbilical artery after the umbilical cord was clamped and cut.

### Immunohistochemistry

Placenta samples from the maternal region were immersed in a 10% neutral formaldehyde solution. About 4–6 m paraffin slices were cut according to the standard paraffin process. The antigen retrieval procedure was carried out twice in citrate buffer solution (pH: 6.0). Endogenous peroxidase activity was inhibited in a 10% hydrogen peroxide solution for 7 min. Before the application of primary antibodies overnight, Ultra V block was applied for 8 min. Then, a secondary antibody was used. The sections were exposed to streptavidin peroxidase for 20 min. The chromogen utilized was diaminobenzidine. The slides were mounted following counterstaining with hematoxylin, washing in tap water for 3 min, and mounting for 2–3 min.

### Western blot analysis

The placenta was ground into a fine powder in a chilled mortar in the presence of liquid nitrogen. Then, cold RIPA buffer containing protease-phosphatase inhibitor cocktail and nuclease was added to milled sample. Also, 20 μg of protein were separated by 10% SDS-PAGE gel and transferred into polyvinyl difluoride membrane (Santa Cruz). Membranes were blocked with 5% skim milk/PBS-T, prior to overnight incubation with anti-SIRT1 (Biolegend) and anti-β-actin (Biolegend) antibodies in 2% skim milk/PBS-T. A suitable HRP-conjugated secondary antibody (Advansa) was utilized to visualize the specific bands. β-Actin was used as a loading control.

## ELISA

The whole blood was gathered and blood was left to clot at 20-25°C for 15–30 min for ELISA. The clot was skimmed by centrifuging at 1,000–2,000 x g for 10 min at +4°C. Separated serums were stored at −80°C. Serum levels of SIRT1 were determined by a human ELISA according to the manufacturer's instructions (SunRed bio, Shanghai, PR China).

### Statistical methods

The statistical analyses were performed using SPSS statistical programming. Data are presented as mean±sd. Normally distributed data in the comparisons between multiple groups were analyzed with a one-way ANOVA. p-value <0.05 was considered to be statistically significant.

## RESULTS

### Protein levels of sirtuins 1 in maternal and umbilical cord serums

A total of 30 individuals in control (30 maternal and 30 umbilical cord serums), 20 patients in the PP group (20 maternal and 20 umbilical cord serums), and 30 patients in the PAS group (30 maternal and 30 umbilical cord serums) were included in this study. Our result showed that there was no statistical difference between SIRT1 levels in both maternal serum samples of control (n=30, mean: 3.8 ng/mL,±sd: 2.0), PP (n=20, mean: 4.4 ng/mL,±sd: 0.6), and PAS (n=30, mean: 4.4 ng/mL,±sd: 3.0) (p=0.299) (Table 1) and umbilical cord serum samples of control (n=30, mean: 6.3 ng/mL,± sd: 3.5), PP (n=20, mean: 6.1 ng/mL,±sd: 2.2), and PAS (n=30, mean: 8.0 ng/mL, ±sd: 4.7) (p=0.135) (Table 1).

**Table 1:** Demographic characteristics, and maternal and fetal serum levels of sirtuins 1 in control, placenta previa, and placenta accreta spectrum groups.

		Control	PP	PAS	p-value
Age (years)		33.3±4.8	33.4±6.0	33.9±4.9	0.883
Gravidity		4.7±1.9	3.2±2.3	5±1.8	*0.009
Parity		3.2±1.4	1.45±1.5	3.3±1.7	**0.0002
Previous cesarean section		2.7±1.1	0	2.3±1.0	–
Birth weight (g)		3076±420	2954±574	2788±430	0.062
Interpregnancy interval (months)					
	≤9	2	0	1	
	>9	28	20	29	
Birth week		37.2±1.2	36.5±2.4	36.3±1.2	0.106
Maternal serum level of SIRT1 (ng/mL)		3.8±2.0	4.4±0.6	4.4±3.0	0.299
Umbilical cord serum level of SIRT1 (ng/mL)		6.3±3.5	6.1±2.2	6.3±3.5	0.135

One-way ANOVA was used for comparison of the three groups.

* p<0.05,

** p<0.01.

### Total protein levels of sirtuins 1 in placenta samples

**Figure 1:**
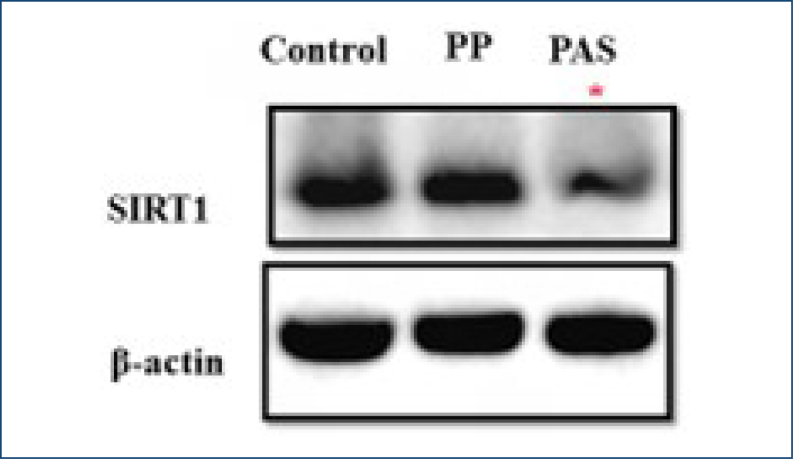
Expression level of sirtuins 1 in placentas of control, placenta previa, and placenta accreta spectrum. Protein levels of sirtuins 1 were determined by Western blotting in the control (n=10), placenta previa (n=10), and placenta accreta spectrum (n=10) groups. β-Actin was used as a loading control. The image indicates a single representative example of experiments (*p<0.05).

The presence of SIRT1 was examined by Western blot analysis in the placental tissues of the three groups via using anti-SIRT1 primary antibodies. Our result showed that SIRT1 expression levels were significantly decreased in PAS patients compared to the control and PP groups (*p<0.037, p<0.05) (Figure 1).

### Sirtuins 1 localization in placenta samples

**Figure 2:**
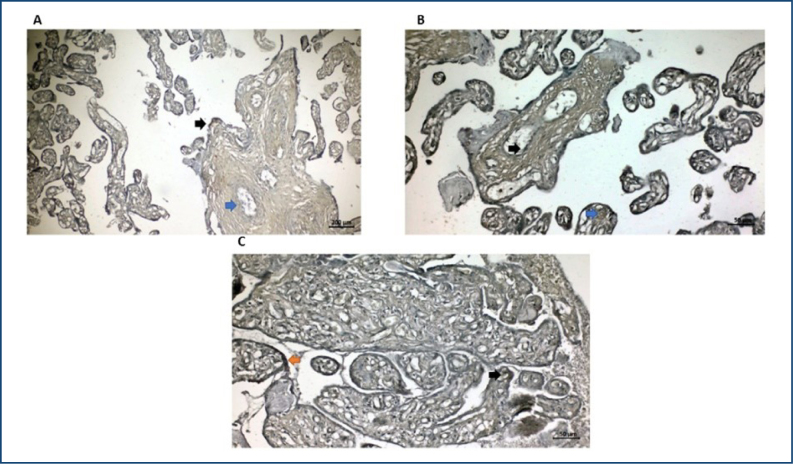
SIRT-1 immunohistochemical staining in the placenta structure of the control (A), placenta previa (B), and placenta accreta spectrum (C) groups. (A) Weakly positive SIRT-1 expression in syncytiotrophoblast cells (black arrow) and negative SIRT-1 expression in fetal capillary endothelium (blue arrow) were observed (bar: 200 μm). (B) Weakly positive SIRT-1 expression in fetal capillary endothelium (black arrow) and positive SIRT-1 expression in villus mesenchyme (blue arrow) were observed (bar: 50 μm). (C) Positive SIRT-1 expression in fetal capillary endothelium (black arrow) and positive SIRT-1 expression in syncytiotrophoblast cells (orange arrow) were observed (bar: 50 μm).

It was observed that SIRT1 expression was weak positive in cytotrophoblast of the control, PP, and PAS groups (Figures 2A, B, C, respectively) in immunohistochemically stained sections. It was also observed that SIRT1 expression was increased specifically in fetal capillary endothelium and syncytiotrophoblast cells in the PAS group but weak positive in villus mesenchyme (Figure 2C). In addition, there was positive expression of SIRT1 in both syncytiotrophoblast and villus mesenchyme but weak positive expression in fetal capillary endothelium of the PP group (Figure 2B). In the control group, weak positive expression in syncytiotrophoblast cells, positive expression in villus mesenchyme, and negative expression in fetal capillary endothelium were observed (Figure 2A).

## DISCUSSION

The increasing number of cases, blood losses, and maternal mortality rates related to PAS reveal the need for urgent diagnosis and treatment methods for PAS. Although uterine scar, cesarean history, and presence of PP are among the clinical risk factors^2^, studies on molecular mechanisms responsible for pathophysiology of PAS are very limited. A few studies have shown that EMT is effective in PAS^14^. Although negative regulation of cell migration was downregulated^15^ and EMT markers are high in PAS^16–18^, there are no studies on the role of SIRT1, which has proven efficacy in cell migration and EMT, in the pathogenesis of the disease. Therefore, we aimed to investigate SIRT1 expression level in PAS.

EMT is a process of molecular and phenotypic epithelial cell replacement that supports invasiveness, resulting in the transformation of immobile epithelial cells into migrant mesenchymal cells^16^. It is activated by epigenetic regulators and shows that abnormally aggressive EMT that continues throughout pregnancy plays a significant role in PAS development^14^. Duzyj et al. observed the co-expression of cytokeratin-7 and vimentin in the PAS group that implies EVTs show their EMT characteristics in the third trimester^16^. Increasing expressions of matrix metalloproteinases (MMP-9 and MMP-2), which play an important role in the penetration of trophoblast cells, were found in samples with PAS^17^. The best characterized event occurring in EMT is the loss of the basic cell–cell adhesion protein E-cadherin. In accretated placenta, the expression of E-cadherin was lower in the chorionic villi of the invasive part, whereas the expression of Snail and TGF-β in the decidual cells of the invasive part increased^19^. In another study, the expression level of ZEB1, the EMT promoter, was found to be high in PAS^9^. It has also shown that methyl-CpG-binding domain protein 2 (MeCP2) regulated by SIRT1, podocin (PODN), and apolipoprotein D (ApoD), which participate in negative regulation of cell migration, were downregulated at the mRNA and protein levels in the PAS group^15^.

SIRT1 governs a variety of cellular processes. In particular, it has been shown to affect epithelial plasticity by reprogramming transcription at the epithelial–mesenchymal transition, leading to invasion and metastasis. SIRT1 controls trophoblast cell invasion. It has been found that SIRT1 knockdown induces a more invasive phenotype in Swan 71 cells accompanied by reduction in proliferation and enhancement of MMP-2 and MMP-9^13^, which overexpressed in PAS, thereby promoting invasion and migration. Moreover, increased invasion resulted from the induction of EMT markers such as N-cadherin, Snail, and ZEB1 and activation of Akt /p38MAPK signaling pathways^13^ as shown in PAS^18^. In addition, a recent study showed that heat shock 70 kDa protein 4 (HSPA4) regulated by SIRT1^19^ is elevated in PAS^20^.

## CONCLUSION

Reduced expression of the SIRT1 in placenta of PAS may be effective in the pathophysiology of PAS. However, further studies need to be conducted to clarify the role of SIRT1 in PAS.

## References

[1] Cahill AG, Beigi R, Heine RP, Silver RM (2018). Placenta accreta spectrum. Am J Obstet Gynecol.

[2] Jauniaux E, Collins S, Burton GJ (2018). Placenta accreta spectrum: pathophysiology and evidence-based anatomy for prenatal ultrasound imaging. Am J Obstet Gynecol.

[3] Silver RM, Branch DW (2018). Placenta accreta spectrum. N Engl J Med.

[4] Gelany S, Mosbeh MH, Ibrahim EM, Mohammed M, Khalifa EM, Abdelhakium AK (2019). Placenta accreta spectrum (PAS) disorders: incidence, risk factors and outcomes of different management strategies in a tertiary referral hospital in Minia, Egypt: a prospective study. BMC Pregnancy Childbirth.

[5] Jauniaux E, Ayres-de-Campos D, Langhoff-Roos J, Fox KA, Collins S (2019). FIGO classification for the clinical diagnosis of placenta accreta spectrum disorders. Int J Gynaecol Obstet.

[6] Einerson BD, Comstock J, Silver RM, Branch DW, Woodward PJ, Kennedy A (2020). Placenta accreta spectrum disorder: uterine dehiscence, not placental invasion. Obstet Gynecol.

[7] Michan S, Sinclair D (2007). Sirtuins in mammals: insights into their biological function. Biochem J.

[8] Palmirotta R, Cives M, Della-Morte D, Capuani B, Lauro D, Guadagni F (2016). Sirtuins and cancer: role in the epithelial-mesenchymal transition. Oxid Med Cell Longev.

[9] Lappas M, Mitton A, Lim R, Barker G, Riley C, Permezel M (2011). SIRT1 is a novel regulator of key pathways of human labor. Biol Reprod.

[10] Broady AJ, Loichinger MH, Ahn HJ, Davy PM, Allsopp RC, Bryant-Greenwood GD (2017). Protective proteins and telomere length in placentas from patients with pre-eclampsia in the last trimester of gestation. Placenta.

[11] Qiao L, Guo Z, Bosco C, Guidotti S, Wang Y, Wang M (2015). Maternal high-fat feeding increases placental lipoprotein lipase activity by reducing SIRT1 expression in mice. Diabetes.

[12] Xiong L, Ye X, Chen Z, Fu H, Li S, Xu P (2021). Advanced maternal age-associated SIRT1 deficiency compromises trophoblast epithelial-mesenchymal transition through an increase in vimentin acetylation. Aging Cell.

[13] Lee KM, Seo HW, Kwon MS, Han AR, Lee SK (2019). SIRT1 negatively regulates invasive and angiogenic activities of the extravillous trophoblast. Am J Reprod Immunol.

[14] Zhu JY, Pang ZJ, Yu YH (2012). Regulation of trophoblast invasion: the role of matrix metalloproteinases. Rev Obstet Gynecol.

[15] Li N, Hou R, Liu C, Yang T, Qiao C, Wei J (2021). Integration of transcriptome and proteome profiles in placenta accreta reveals trophoblast over-migration as the underlying pathogenesis. Clin Proteomics.

[16] Duzyj CM, Buhimschi IA, Motawea H, Laky CA, Cozzini G, Zhao G (2015). The invasive phenotype of placenta accreta extravillous trophoblasts associates with loss of E-cadherin. Placenta.

[17] Demir-Weusten AY, Seval Y, Kaufmann P, Demir R, Yucel G, Huppertz B (2007). Matrix metalloproteinases-2, −3 and −9 in human term placenta. Acta Histochem.

[18] Li N, Yang T, Yu W, Liu H, Qiao C, Liu C (2019). The role of Zeb1 in the pathogenesis of morbidly adherent placenta. Mol Med Rep.

[19] Yang Y, Zhang S, Guan J, Jiang Y, Zhang J, Luo L (2022). SIRT1 attenuates neuroinflammation by deacetylating HSPA4 in a mouse model of Parkinson's disease. Biochim Biophys Acta Mol Basis Dis.

[20] Li SC, Lan KC, Hung HN, Huang WT, Lai YJ, Cheng HH (2022). HSPA4 is a biomarker of placenta accreta and enhances the angiogenesis ability of vessel endothelial cells. Int J Mol Sci.

